# Die Mikroarchitektur des Pankreaskarzinoms aus Sicht des Pathologen und des Radiologen

**DOI:** 10.1007/s00292-021-00949-2

**Published:** 2021-05-06

**Authors:** Philipp Mayer, Matthias M. Gaida

**Affiliations:** 1 Klinik für diagnostische und interventionelle Radiologie, Universitätsklinikum Heidelberg, Im Neuenheimer Feld 420, 69120 Heidelberg, Deutschland; 2 Institut für Pathologie, Universitätsmedizin, JGU Mainz, 55131 Mainz, Deutschland; 3 Joint Unit Immunpathologie, Translationale Onkologie, Institut für Pathologie, Universitätsmedizin Mainz, 55131 Mainz, Deutschland

**Keywords:** Pankreaskarzinom, Radiologie, Pathologie, Duktales Adenokarzinom des Pankreas, Magnetresonanztomographie, Computertomographie, Pancreatic cancer, Radiology, Pathology, Pancreatic adeno carcinoma, Magnetic resonance imaging, Computed tomography

## Abstract

Die diagnostische Radiologie ist gemeinsam mit der diagnostischen Pathologie eines der klinisch-morphologischen Fächer, welche in unterschiedlicher makroskopischer bzw. mikroskopischer Auflösung zur Detektion, Charakterisierung sowie zum Ausbreitungsmuster eines Tumors führen. Die klinischen Disziplinen sind oft voneinander getrennt, wenngleich es vor allem in klinischen Tumorboards immer stärkere Verzahnungen gibt. Am Beispiel des Pankreaskarzinoms sind die Korrelationen radiologischer und pathologischer Diagnostik dargestellt.

## Abstract (en)

Diagnostic radiology and diagnostic pathology are medical disciplines that use a variety of morphological analyses with different macroscopic and microscopic resolutions for diagnosis and staging of cancers. In the clinical setting, radiology and pathology departments are often spatially separated. However, there are examples of increasingly tight cooperation and convergence, for example in the setting of multidisciplinary tumor boards. This article focuses exemplarily on the correlations of radiological and histopathological diagnostics in pancreatic cancer.

## Radiologische Charakterisierung des Pankreaskarzinoms

Die Radiologie spielt eine wichtige Rolle bei der initialen Diagnosestellung, Ausbreitungsdiagnostik und Therapie‑/Operationsplanung des duktalen Adenokarzinom des Pankreas (PDAC) gefolgt von der weiterführenden ultrastrukturellen Aufarbeitung durch den Pathologen. Die Kombination der radiologischen und pathologischen Befunde, naturgegeben oftmals zusammen mit dem intraoperativen Situs und weiteren diagnostischen Laborparametern, ergeben ein Gesamtbild über den Typus, die Ausbreitung und die Biologie der Tumorerkrankung. In interdisziplinären Tumorboards werden auf Basis der Zusammenschau der in den verschiedenen Disziplinen erhobenen Befunde konsensuale Therapieentscheidungen getroffen.

**Figure 1:**
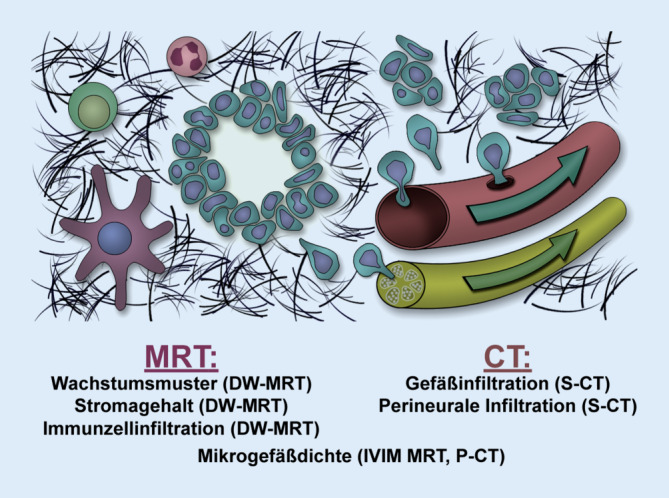

Die meisten PDAC erscheinen radiologisch als unscharf begrenzte minderkontrastierte solide Raumforderungen mit infiltrativem Ausbreitungsmuster. Die kontrastmittelgestützte Computertomographie (CT) und Magnetresonanztomographie (MRT) können mit hoher Genauigkeit PDACs diagnostizieren [27]. Während die MRT aufgrund ihres besseren Weichteilkontrastes Vorteile bei der Detektion von Lebermetastasen hat, ist die CT die meistangewandte und bestvalidierte radiologische Methode zur Beurteilung der Ausdehnung, des Gefäßbezuges und damit der Resektabilität [21, 31]. Auch die extrapankreatische perineurale Invasion lässt sich mit der CT gut vorhersagen [12]. Neben dieser Darstellung der makroskopischen Tumorausdehnung vermögen moderne funktionelle Bildgebungstechniken Mikrostrukturparameter der abgebildeten Gewebe abzuschätzen (Abb. 1), wenngleich die direkte In-vivo-Darstellung einzelner Tumorzellen oder Bestandteile des Stromas mittels nichtinvasiver CT- und MRT-Bildgebung (noch) nicht möglich ist und bisher einzig der mikroskopischen Diagnostik durch den Pathologen vorbehalten ist. Eine gut charakterisierte funktionelle radiologische Untersuchungsmethode ist die kontrastmittelgestützte CT-Perfusion, die zur quantitativen Bestimmung der Gewebedurchblutung verwendet wird. Die CT-Perfusion kann die Detektionsrate von PDACs im CT zu verbessern, da sich das Pankreaskarzinom aufgrund seiner geringen Perfusion als minderdurchblutetes Areal abzeichnet [15]. Eine weitere etablierte funktionelle radiologische Untersuchungsmethode, die diffusionsgewichtete MRT (DW-MRT), kommt ohne Kontrastmittelapplikation und Röntgenstrahlung aus. Die DW-MRT quantifiziert die wärmeinduzierte Bewegung von Wassermolekülen in menschlichen Geweben. Die Diffusion wird klinisch meist durch den sog. apparenten Diffusionskoeffizienten (ADC) quantifiziert, dessen Berechnung ein monoexponentielles Diffusionsmodell zugrunde liegt. Sie ist meist stärker eingeschränkt in kompakten Geweben, die viele mikrostrukturelle Hindernisse (z. B. Zellmembranen bei erhöhter Zelldichte) für die freie Wasserdiffusion aufweisen. Hierzu zählen auch die meisten malignen Tumoren [2]. Auch in den meisten PDACs ist die Diffusion stärker eingeschränkt als im nichtneoplastischen Pankreasparenchym, was zur Detektion der Tumoren nützlich sein kann [2]. Ein Nachteil des monoexponentiellen Diffusionsmodelles ist, dass die ermittelten ADC-Werte auch durch Perfusionseffekte im Gewebe beeinflusst werden. Le Bihan entwickelte daher das biexponentielle Intravoxel-Incoherent-Motion(IVIM)-DW-MRT-Modell, welches eine Trennung von Diffusions- und Perfusionseffekten ermöglicht [2].

## Korrelation von Histologie und Radiologie

Das Pankreaskarzinom erscheint am Operationspräparat makroskopisch in der Regel als derber, solider, unscharf begrenzter Tumor, was oftmals schon rein bildmorphologisch mit den Befunden der CT oder MRT korreliert. Mikroskopisch ist das PDAC aufgebaut aus atypisch konfigurierten Drüsen, umgeben von einem desmoplastischen Stroma, welches den Großteil des Tumorvolumens einnehmen kann [4]. Neben dem prädominanten drüsigen Wachstum, sind auch (mikro-)papilläre, solide-nestartige, kribriforme oder einzelzellig-dissoziierte Muster nachweisbar [19]. Verschiedene Faktoren, wie beispielsweise ein mesenchymaler Tumorphänotyp, sind mit einem einzelzellig-dissoziierten bzw. histologisch schlecht differenzierten Tumorwachstumsmuster assoziiert [7]. Im Gegensatz zur lobulären Anordnung der Drüsen im gesunden Pankreas sind die Tumordrüsen diffus im Stroma angeordnet, deformiert und weisen unvollständige Lumina auf bzw. die Zellen vereinzeln sich [25]. Typisch ist ein dispergiertes Wachstumsmuster, d. h., die Tumorzellen wachsen häufig nicht als zusammenhängende Tumormasse, sondern man findet Zellcluster, die einen erheblichen Abstand zur Haupttumormasse aufweisen und umliegendes Gewebe, Nervenscheiden und Gefäße infiltrieren [29]. Als charakteristisch für das PDAC gilt eine inter- und intratumorale Heterogenität hinsichtlich des Wachstumsmusters, der zytologischen Charakteristika und der Stromaeigenschaften. Zytogenetische Heterogenität wurde sogar zwischen unmittelbar aneinander grenzenden Tumordrüsen berichtet [29]. Das histologische Wachstumsmuster beeinflusst die radiologische Darstellung in der MRT entscheidend. PDAC mit primär glandulärem Wachstumsmuster weisen höhere ADC-Werte auf als solide, einzelzellig oder mikropapillär wachsende PDACs [19]. Zu erklären ist diese Beobachtung damit, dass luminale Strukturen eine freiere Wasserdiffusion ermöglichen verglichen mit soliden Zellverbänden oder gar einzelliegenden Tumorzellen, wo es kaum oder keine freien Lumina gibt. Hierdurch wird das Gewebe deutlich kompakter und somit schränken diese mikrostrukturellen Hindernisse die freie Wasserdiffusion ein.

Palpatorisch sind PDAC meist derb, was primär auf ihren hohen Anteil an kollagenreichem Stroma (sog. desmoplastische Reaktion) zurückzuführen ist [22]. Das Stroma, welches histologisch bis zu 90 % der Tumormasse ausmacht, setzt sich aus der extrazellulären Matrix (EZM) sowie zahlreichen unterschiedlichen Zelltypen zusammen. Die EZM ist ein komplexes Netzwerk von Makromolekülen, welches zum einen als Gerüst eine Stützfunktion für den Tumor ausübt und zum anderen durch unterschiedliche biochemische Signalwege die Tumorzellen wesentlich beeinflusst [16]. Hauptbestandteil der EZM sind Kollagene, die nicht nur quantitativ vermehrt vorliegen, sondern auch qualitativ gegenüber dem nichtneoplastischen Pankreasparenchym verändert sind [30]. Durch Fehlen der regelhaften Strukturen der Basalmembran erhalten die Kollagene einen alterierten Kontakt zu PDAC-Zellen und interagieren mit ihnen [30]. Zahlreich findet man im Stroma auch Proteoglykane, die verantwortlich sind für den hohen interstitiellen Flüssigkeitsdruck der PDACs, der durch Kompression der Tumorgefäße zu einer geringen Durchblutung der Tumoren beiträgt [11].

**Figure 2:**
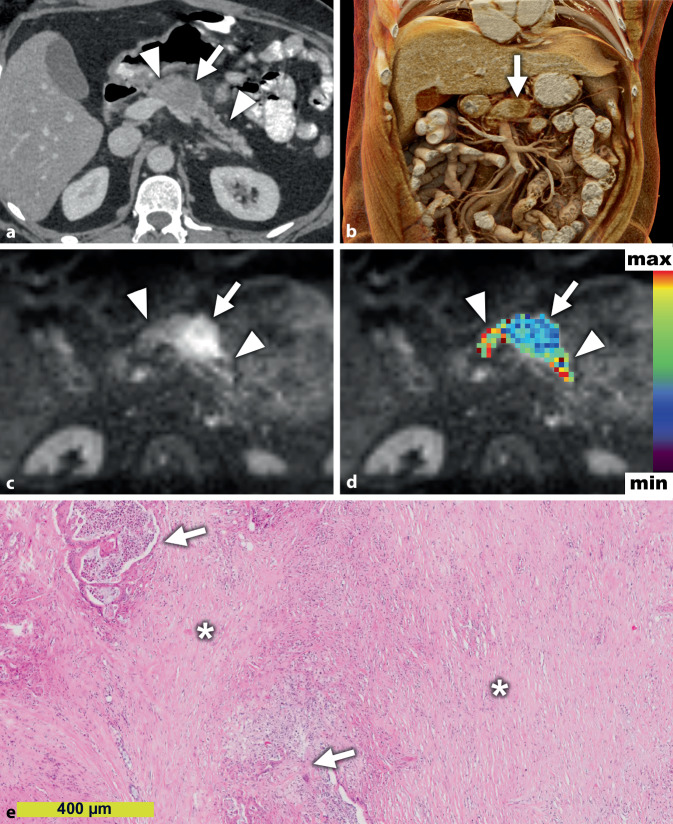

Der Stromagehalt ist neben der Zelldichte [10] und dem Wachstumsmuster [19] einer derjenigen Mikrostrukturparameter, die maßgeblich die Diffusion in der DW-MRT des PDAC beeinflussen [23]. Die Makromoleküle der EZM stellen physikalische Barrieren für die freie Diffusion von Wassermolekülen dar, weshalb die Diffusion in stromareichen PDACs stärker eingeschränkt ist als in stromaarmen PDACs (Abb. 2 und 3; [22]). So könnte die Diffusionsbildgebung in Zukunft bei der Therapiestratifizierung und dem Therapiemonitoring von Patienten für antistromale Therapien hilfreich sein. Die Zusammensetzung des Stromas, beispielsweise durch eine Vermehrung von proteaseproduzierenden Myofibroblasten, deren Aktivitätsstatus sowie die Infiltration von (proteaseproduzierenden) Immunzellen, bestimmt somit folglich die Mikroarchitektur des Stromas des PDAC und konsequenterweise die Diffusion [7, 19, 24]. Wie auch für Prostatakarzinome und Kopf-Hals-Tumoren beschrieben, ist die Diffusion häufig stärker eingeschränkt in PDACs mit ausgeprägter und durch immunhistochemische Marker verifizierter Tumorhypoxie, die durch den oftmals kompakteren Gewebeaufbau eine deutliche Minderperfusion mit Sauerstoff aufweisen, als PDACs mit geringer Tumorhypoxie [23]. Dies ist potenziell therapeutisch relevant, da hypoxische Tumoren resistenter gegenüber Strahlentherapie sein können, und steht in Einklang mit der Beobachtung, dass PDACs mit niedrigeren ADC-Werten vor Therapie häufig schlechter auf eine neoadjuvante Radiochemotherapie ansprechen als Patienten mit hohen ADC-Werten [3].

**Figure 3:**
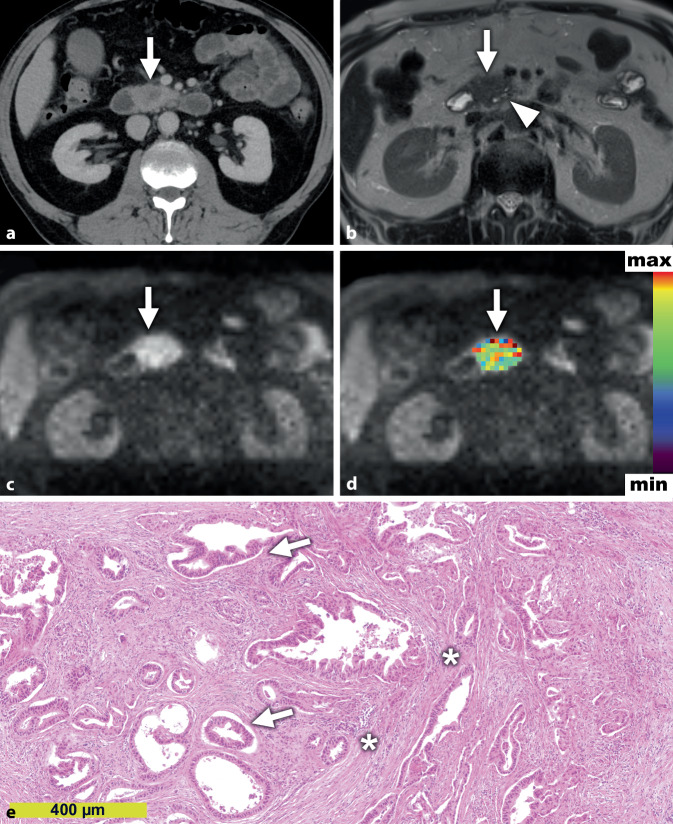

Die mit dem IVIM-DW-MRT-Modell berechnete Perfusionsfraktion *f* vermag ähnlich gut wie die CT-Perfusion die Durchblutung und histopathologisch quantifizierte Mikrogefäßdichte des PDAC abzuschätzen [14, 20]. Für die klinische Praxis ist relevant, dass die Perfusionsfraktion *f* aus dem biexponentiellen IVIM-Modell eine höhere diagnostische Güte für die Differenzierung zwischen PDACs und chronischen Pankreatitiden aufweist als der ADC aus dem monoexponentiellen Diffusionsmodell [13].

Inwiefern das Stroma das Tumorwachstum hindert oder fördert, ist zurzeit Gegenstand intensiver Forschung und kontrovers diskutiert [11]. Lange Zeit hatte man die EZM mit ihrem dichten Kollagennetzwerk für eine Abwehrreaktion des Körpers gehalten, um das Tumorwachstum des PDAC einzudämmen [11]. Unterstützt wurde diese Theorie durch In-vitro-Experimente, in welchen ein inhibitorischer Effekt von Fibroblasten auf verschiedene Tumorzelllinien gezeigt wurde [11]. Für Laminin, ein Glykoprotein der EZM wurde eine hemmende Wirkung auf die die Tumorprogression gezeigt [30]. Mittlerweile weiß man jedoch, dass viele Bestandteile des Stromas eine fördernde Rolle bei der Tumorprogression spielen. Die Steifigkeit des Stromas kann die Polarität der Tumorzellen reduzieren und die epitheliale zu mesenchymale Transition (EMT) induzieren [30]. Kollagene können die Proliferation und Migration der Tumorzellen fördern [8] und diverse Matrixmetalloproteinasen (MMP) vermitteln die hierfür nötige Stromaplastizität [30]. Das Stroma wirkt außerdem als eine Art physikalische und biochemische Barriere, die die Wirksamkeit von Radiochemotherapien einschränken kann [16]. Insgesamt scheinen die protumorigenen Eigenschaften des Stromas zu überwiegen, da ein hoher Stromaanteil mit einer schlechteren Prognose assoziiert ist [11].

Aus diesem Grund wurde initial große Hoffnung auf neue Therapien, die die Tumor-Stroma-Interaktionen angreifen, gesetzt [11]. Auf vielversprechende Ergebnisse initialer präklinischer Studien folgte schließlich die Ernüchterung, als weiterführende Studien zeigten, dass eine komplette Depletion des Stromas zu aggressiveren Tumoren führt und die Metastasierung begünstigt [28]. Daher haben neuere Therapieansätze meist nicht mehr die komplette Depletion des Stromas, sondern vielmehr die Wiederherstellung der Gewebehomöostase im Stroma oder Modulation des Stromas zum Ziel [28]. Die Identifikation und Validierung einer histologischen Signatur der zellulären und azellulären Bestandteile des Stromas schafft die Basis für die Entwicklung dieser Therapien [18]. Die radiologische Evaluation stellt eine dynamische Ergänzung zum Therapiemonitoring und zur vorausgegangenen Biopsie dar.

Im Stroma finden sich verschiedene Zellarten, unter anderem regulatorische und zytotoxische Immunzellen, Endothelzellen, Neuronen sowie Sternzellen bzw. aktivierte Myofibroblasten [11]. Letztere sind verantwortlich für die Produktion eines Großteils der extrazellulären Matrix [30] und wirken protumorigen, unter anderem durch Förderung der Tumorzellproliferation [11]. Der Hauptteil der nichtepithelialen Zellen im Stroma sind Immunzellen [11]. Die Immunzellen erzeugen durch die Produktion von Chemokinen, Zytokinen, Wachstumsfaktoren und Proteasen ein fibroinflammatorisches Milieu, welches maßgeblich zur Progression des PDAC beiträgt [5]. Die Zusammensetzung des Immuninfiltrates moduliert die Mikroarchitektur des Pankreaskarzinoms und somit folglich das histologische Bild und die Darstellung in der Radiologie. Neutrophile Granulozyten modifizieren die Stromazusammensetzung durch eine Reprogrammierung der myofibroblastären Zellen mittels Induktion u. a. von MMP9 und beeinflussen somit indirekt das Wachstumsmuster des PDAC zu einem nichtdrüsigen Phänotyp [19]. Außerdem induzieren Neutrophile mittels Elastase, durch die proteolytische Spaltung des Zelladhäsionsmoleküls E‑Cadherin, in den Tumorzellen eine EMT und eine histologisch sichtbare Tumorzellvereinzelung [6, 7]. Folglich zeigen neutrophilenreiche Pankreaskarzinome durch ihr alteriertes, nichtdrüsiges Wachstumsmuster und der somit histologisch evidenten kompakten Architektur ein Einschränkung der Wasserdiffusion mit niedrigeren ADC-Werten verglichen zu den konventionellen, drüsig wachsenden PDAC, wo signifikant höhere ADC-Werte gemessen werden [19].

Neben neutrophilen Granulozyten konnte auch zu intratumoralen T‑Zellen, eine der prävalentesten Immunzellen im PDAC, eine Assoziation zur Bildgebung hergestellt werden. Die über einen alternativen p38-MAP-Kinase-Signalweg aktivierten CD4+-T-Zellen zeigen eine Th17-Signatur und sind mit einer höheren Gefäßdichte in PDAC und schlechterer Prognose assoziiert [1]. Tumoren mit einem dichten Infiltrat dieser Th17-artigen CD4+-T-Zellen sind häufig mit einer alterierten Wasserdiffusion in der MRT assoziiert verglichen zu den lymphozytenarmen Tumoren[24].

## Ausblick

Zusammenfassend gibt es in der Radiologie einen zunehmenden Trend zu Quantifizierung von Bildmerkmalen über die reine Morphologie hinaus. Die automatische Extraktion und Analyse einer großen Zahl von Bildmerkmalen, die teilweise für das bloße Auge unsichtbar sind, wird als Radiomics bezeichnet. Obwohl die Radiomicsforschung noch in den Anfängen steckt [9], ist es nur eine Frage der Zeit, bis sie Einzug in die klinische Routine erhält. Analysealgorithmen, wie sie Radiomics zugrunde liegen, sind prinzipiell nicht nur auf radiologische, sondern auch auf histopathologische Bilddaten und genomische Daten anwendbar. Begünstigt auch durch die beginnende Umstellung der pathologischen Diagnostik von der analogen, slidebasierten Mikroskopie zu einem digitalen Workflow ermöglicht dies eine noch engere Verzahnung von Pathologie und Radiologie (sog. Integrated Diagnostics), zum Beispiel durch Speichern der histopathologischen und radiologischen Bilddaten in einem gemeinsamen Bildarchivierungssystem [17]. Dies verdeutlicht, dass Pathologie und diagnostische Radiologie im Grunde stark miteinander verknüpft sind, da sie medizinische Informationen aus Bilddaten extrahieren und interpretieren. Radiologisch-pathologische Korrelationen sind in der klinischen Routine essenziell, um Fehlentscheidungen zu vermeiden. Sie helfen beispielsweise Samplingfehler bei Probeentnahmen aus Tumoren mit einem hohen Grad an Heterogenität (wie dem PDAC) oder Fehlbiopsien zu erkennen und korrekt zu interpretieren. So wird in Zeiten der personalisierten Therapie neben den klinischen und laborchemischen Parametern insbesondere auch eine Integration der radiologischen und (histo‑)pathologischen Parameter zum besseren Verständnis von (Pankreas‑)Karzinompatienten vorgeschlagen [26].

## Fazit für die Praxis

- Das duktale Adenokarzinom des Pankreas (PDAC) zeichnet sich histologisch durch ein hohes Maß an intratumoraler Heterogenität, einen hohen Stromaanteil mit typischer desmoplastischer Reaktion sowie ausgeprägtes Entzündungszellinfiltrat, ein infiltratives und dissoziiertes Wachstumsmuster und relativ geringe Tumorperfusion aus.
- Viele histologisch sichtbare Mikrostruktureigenschaften des PDAC lassen sich durch funktionelle radiologische Untersuchungsmethoden wie die CT-Perfusion und die diffusionsgewichtete MRT (DW-MRT) abschätzen.
- Die funktionelle Bildgebung, die Strukturen und Prozesse auf subzellulärer Ebene zu quantifizieren vermag, könnte im Zeitalter der personalisierten Medizin eine zunehmend wichtigere Rolle bei der Tumorcharakterisierung und dem Therapiemonitoring von PDAC-Patienten einnehmen und stellt somit die offenkundig doch enge Verzahnung der Radiologie und Pathologie dar.

## References

[1] Alam MS, Gaida MM, Bergmann F (2015). Selective inhibition of the p38 alternative activation pathway in infiltrating T cells inhibits pancreatic cancer progression. Nat Med.

[2] Barral M, Taouli B, Guiu B (2015). Diffusion-weighted MR imaging of the pancreas: current status and recommendations. Radiology.

[3] Cuneo KC, Chenevert TL, Ben-Josef E (2014). A pilot study of diffusion-weighted MRI in patients undergoing neoadjuvant chemoradiation for pancreatic cancer. Transl Oncol.

[4] Erkan M, Reiser-Erkan C, Michalski CW (2009). Cancer-stellate cell interactions perpetuate the hypoxia-fibrosis cycle in pancreatic ductal adenocarcinoma. Neoplasia.

[5] Felix K, Gaida MM (2016). Neutrophil-derived proteases in the microenvironment of pancreatic cancer—active players in tumor progression. Int J Biol Sci.

[6] Gaida MM, Steffen TG, Günther F (2012). Polymorphonuclear neutrophils promote dyshesion of tumor cells and elastase-mediated degradation of E-cadherin in pancreatic tumors: innate immunity. Eur J Immunol.

[7] Große-Steffen T, Giese T, Giese N (2012). Epithelial-to-mesenchymal transition in pancreatic ductal adenocarcinoma and pancreatic tumor cell lines: the role of neutrophils and neutrophil-derived elastase. Clin Dev Immunol.

[8] Grzesiak JJ, Ho JC, Moossa AR, Bouvet M (2007). The integrin-extracellular matrix axis in pancreatic cancer. Pancreas.

[9] He M, Xue H, Jin Z (2020). Radiomics in pancreatic ductal adenocarcinoma: a state of art review. Int. J. Pancreatol..

[10] Heid I, Steiger K, Trajkovic-Arsic M (2017). Co-clinical assessment of tumor cellularity in pancreatic cancer. Clin Cancer Res.

[11] Hessmann E, Buchholz SM, Demir IE (2020). Microenvironmental determinants of pancreatic cancer. Physiol Rev.

[12] Khristenko E, Shrainer I, Setdikova G (2021). Preoperative CT-based detection of extrapancreatic perineural invasion in pancreatic cancer. Sci Rep.

[13] Klauß M, Lemke A, Grünberg K (2011). Intravoxel incoherent motion MRI for the differentiation between mass forming chronic pancreatitis and pancreatic carcinoma. Invest Radiol.

[14] Klauß M, Mayer P, Bergmann F (2015). Correlation of histological vessel characteristics and diffusion-weighted imaging Intravoxel incoherent motion-derived parameters in pancreatic ductal adenocarcinomas and pancreatic neuroendocrine tumors. Invest Radiol.

[15] Klauß M, Stiller W, Fritz F (2012). Computed tomography perfusion analysis of pancreatic carcinoma. J Comput Assist Tomogr.

[16] Lafaro KJ, Melstrom LG (2019). The paradoxical web of pancreatic cancer tumor microenvironment. Am J Pathol.

[17] Lundström CF, Gilmore HL, Ros PR (2017). Integrated diagnostics: the computational revolution catalyzing cross-disciplinary practices in radiology, pathology, and genomics. Radiology.

[18] Mahajan UM, Langhoff E, Goni E (2018). Immune cell and stromal signature associated with progression-free survival of patients with resected pancreatic ductal adenocarcinoma. Gastroenterology.

[19] Mayer P, Dinkic C, Jesenofsky R (2018). Changes in the microarchitecture of the pancreatic cancer stroma are linked to neutrophil-dependent reprogramming of stellate cells and reflected by diffusion-weighted magnetic resonance imaging. Theranostics.

[20] Mayer P, Fritz F, Koell M (2021). Assessment of tissue perfusion of pancreatic cancer as potential imaging biomarker by means of Intravoxel incoherent motion MRI and CT perfusion: correlation with histological microvessel density as ground truth. Cancer Imaging.

[21] Mayer P, Giannakis A, Klauß M (2021). Radiological evaluation of pancreatic cancer: what is the significance of arterial encasement 〉180° after neoadjuvant treatment?. Eur J Radiol.

[22] Mayer P, Jiang Y, Kuder TA (2020). Diffusion kurtosis imaging—a superior approach to assess tumor-stroma ratio in pancreatic ductal adenocarcinoma. Cancers.

[23] Mayer P, Kraft A, Witzel HR (2020). Restricted water diffusion in diffusion-weighted magnetic resonance imaging in pancreatic cancer is associated with tumor hypoxia. Cancers.

[24] Mayer P, Linnebacher A, Glennemeier-Marke H (2020). The microarchitecture of pancreatic cancer as measured by diffusion-weighted magnetic resonance imaging is altered by T cells with a tumor promoting th17 phenotype. Int J Mol Sci.

[25] Ren B, Liu X, Suriawinata AA (2019). Pancreatic ductal adenocarcinoma and its precursor lesions. Am J Pathol.

[26] Thompson ED, Roberts NJ, Wood LD (2020). The genetics of ductal adenocarcinoma of the pancreas in the year 2020: dramatic progress, but far to go. Mod Pathol.

[27] Toft J, Hadden WJ, Laurence JM (2017). Imaging modalities in the diagnosis of pancreatic adenocarcinoma: a systematic review and meta-analysis of sensitivity, specificity and diagnostic accuracy. Eur J Radiol.

[28] van Mackelenbergh MG, Stroes CI, Spijker R (2019). Clinical trials targeting the stroma in pancreatic cancer: a systematic review and meta-analysis. Cancers.

[29] Verbeke C (2016). Morphological heterogeneity in ductal adenocarcinoma of the pancreas—does it matter?. Pancreatology.

[30] Weniger M, Honselmann KC, Liss AS (2018). The extracellular matrix and pancreatic cancer: a complex relationship. Cancers.

[31] Zaky AM, Wolfgang CL, Weiss MJ (2017). Tumor-vessel relationships in pancreatic ductal adenocarcinoma at multidetector CT: different classification systems and their influence on treatment planning. Radiographics.

